# Coordination of Lipid Storage and Mobilization Pathways During Osteoblast Maturation in a 3D Human Bone Model

**DOI:** 10.3390/ijms27073325

**Published:** 2026-04-07

**Authors:** Maria Giovanna Rizzo, Dario Morganti, Emanuele Luigi Sciuto, Antonella Smeriglio, Giorgia Cannatà, Barbara Fazio, Salvatore P. P. Guglielmino, Domenico Trombetta, Caterina Faggio, Sabrina Conoci

**Affiliations:** 1 Department of Chemical, Biological, Pharmaceutical and Environmental Sciences (ChiBioFarAm), University of Messina, 98166 Messina, Italy; dario.morganti@unime.it (D.M.); emanueleluigi.sciuto@unime.it (E.L.S.); antonella.smeriglio@unime.it (A.S.); giorgia.cannata@studenti.unime.it (G.C.); salvatore.guglielmino@unime.it (S.P.P.G.); domenico.trombetta@unime.it (D.T.); sabrina.conoci@unibo.it (S.C.); 2 Institute for Microelectronics and Microsystems (CNR IMM-ME), 98166 Messina, Italy; barbara.fazio@cnr.it; 3 Department of Eco-Sustainable Marine Biotechnology, Stazione Zoologica Anton Dohrn, 80122 Naples, Italy; 4 Department of Chemistry “Giacomo Ciamician”, University of Bologna, Via Selmi, 2, 40126 Bologna, Italy

**Keywords:** bone physiology, three-dimensional cell culture, bone tissue model, lipid droplets, lipid metabolism, mitochondrial β-oxidation, osteogenic differentiation, osteoblasts

## Abstract

Bone formation requires a substantial energy supply to sustain extracellular matrix production and mineralization, yet the temporal contribution of lipid metabolism during osteoblast maturation remains incompletely characterized. This study investigated the molecular and transcriptional remodeling of lipid metabolism. Intracellular lipid distribution was analyzed by confocal microscopy using Nile Red staining. Transcriptional modulation of lipid synthesis, storage, lipolysis, genes associated with mitochondrial fatty acid oxidation, and osteogenic markers were assessed by quantitative real-time PCR, and the biochemical composition was evaluated by Raman spectroscopy. Early stages of spheroid development showed higher expression of genes involved in lipid synthesis and storage (FASN, DGAT2, and PLIN2) together with intracellular lipid accumulation, whereas later stages displayed increased expression of lipolytic and β-oxidation markers (PNPLA2/ATGL, CPT1A, and HADHA), accompanied by the redistribution of lipid droplets. The Raman analysis revealed a time-dependent variation of lipid-associated CH_2_/CH_3_ bands and modulation of protein-related Amide I–III signals, consistent with biochemical remodeling during maturation. Overall, the data indicate a coordinated transcriptional shift from lipid accumulation-associated pathways toward lipid mobilization during osteogenic progression in a 3D culture. This model provides a controlled experimental platform for investigating metabolic regulation during bone formation and for studying metabolic alterations associated with skeletal disorders.

## 1. Introduction

Bone is a dynamic tissue that undergoes continuous remodelling to preserve skeletal integrity [1,2,3]. This process relies on the balance between osteoclast-mediated resorption and osteoblast-driven bone formation, maintained through tightly coordinated mechanisms [4,5]. Bone formation is a highly energy-demanding process characterized by a high energetic demand for extracellular matrix (ECM) synthesis and mineralization vesicle production [6]. Osteoblasts require substantial amounts of adenosine triphosphate (ATP) [7], derived from metabolic processes utilizing substrates such as glucose, amino acids, and fatty acids [8]. Although osteoblasts display a predominantly glycolytic phenotype, oxidative metabolism also contributes significantly to ATP production [8,9]. Recent evidence highlights the importance of lipid metabolism in osteoblast bioenergetics. The skeleton has been identified as a site of significant lipoprotein uptake [10], and excess fatty acids are incorporated into intracellular lipid droplets. Importantly, inhibition of lipid droplet formation impairs osteoblast differentiation, supporting their functional relevance [11]. Lipid metabolism in osteoblasts involves a balance between synthesis, storage, and utilization. De novo lipogenesis enables the conversion of carbon substrates into fatty acids, which are stored as triglycerides in lipid droplets. These organelles are metabolically active and interact with mitochondria, allowing rapid mobilization of fatty acids to meet energetic demands [4]. Free fatty acids are then oxidized via mitochondrial β-oxidation and oxidative phosphorylation [11,12]. Thus, lipid metabolism represents a key component of osteoblast differentiation and function rather than a secondary energy source, contributing to metabolic adaptation during differentiation and functional demand [11,12,13]. Furthermore, the spatial organization of lipid droplets appears to be associated with osteoblast cellular architecture and differentiation status [14].

Despite these advances, most knowledge on osteoblast metabolism derives from two-dimensional (2D) culture systems, which present significant limitations. In 2D cultures, cells do not accurately reproduce bone architecture, mechanical cues, or biochemical gradients, leading to altered morphology, gene expression, and metabolic behavior [15]. In particular, metabolic pathways are strongly influenced by cell–cell interactions, oxygen availability, and spatial organization, which are poorly represented in 2D conditions [15].

Three-dimensional (3D) cell culture models have, therefore, emerged as more physiologically relevant systems. In 3D environments, osteoblasts establish complex cell–cell and cell–matrix interactions and are exposed to gradients of nutrients and oxygen that better mimic in vivo conditions [16,17,18]. These features are particularly relevant for studying metabolic regulation, including lipid metabolism [16,19]. Accordingly, 3D osteoblast cultures represent a valuable platform to investigate how lipid metabolic pathways are regulated during bone formation [13,20].

In a previous study [21], a reproducible 3D human osteoblast spheroid model was established and structurally characterized to recapitulate key morphological and extracellular matrix features of bone tissue. Building on this model, the present study investigates the temporal regulation of lipid metabolism during osteogenic differentiation in a 3D microenvironment. Specifically, we evaluate lipid synthesis, lipid droplet dynamics, lipolysis, and mitochondrial β-oxidation during spheroid maturation, correlating transcriptional changes with spatial and biochemical lipid organization.

The aim of this study is to define the contribution of lipid metabolism to osteoblast functional progression in a 3D culture and to determine whether coordinated modulation of lipid-related pathways accompanies extracellular matrix organization and mineralization.

## 2. Results

### 2.1. Subsection

#### Lipid Distribution Assessed by Confocal Microscopy

**Figure 1:**
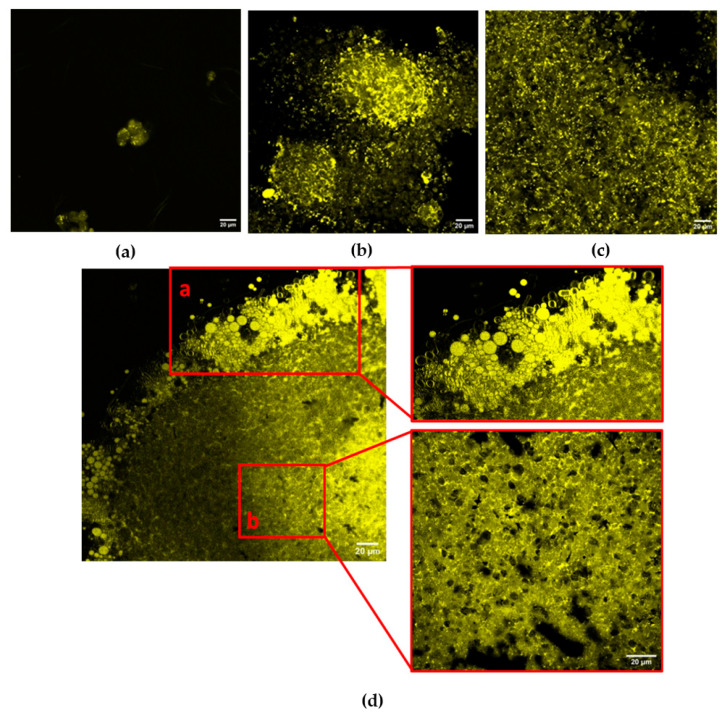
Confocal microscopy images of hFOB spheroid development at 1 day (**a**), 1 week (**b**), 2 weeks (**c**), and 4 weeks (**d**) of culture. Higher magnification views of 4-week spheroids highlight lipid-rich peripheral regions (red box a) and inner core areas (red box b). Nile Red-positive lipid droplets are shown in yellow. Scale bar: 20 µm.

The spheroids were characterized by confocal microscopy, from 1 day to 4 weeks, as shown in Figure 1. Figure 1a shows early aggregates observed after day 1 of growth, characterized by small and dispersed Nile Red-positive lipid vesicles, showing the presence of lipid droplets. After 1 week (Figure 1b), spheroids exhibited cell–cell self-aggregation and a marked increase in lipid signal intensity. Lipid droplets appeared more abundant and mainly concentrated in central regions, suggesting active lipid accumulation during structural consolidation. After 2 weeks (Figure 1c), spheroids showed a higher aggregation level and size, while lipid droplets appeared more uniformly distributed throughout the structure, showing a more homogeneous lipid distribution. After 4 weeks (Figure 1d), a confocal analysis revealed clear spatial compartmentalization of lipid distribution within mature spheroids. Lipid-rich regions were predominantly located at the periphery, where large, intensely fluorescent vesicular structures were observed (Figure 1d), while the inner core showed a trabecular-like organization characterized by smaller, more dispersed lipid droplets (Figure 1d). This spatial heterogeneity indicates stage-dependent redistribution of lipid droplets within the spheroid architecture. These observations provide a qualitative visualization of lipid droplet distribution within the spheroid architecture during maturation.

Control samples processed under identical acquisition conditions, but without Nile Red staining, were examined to assess background fluorescence. Under these conditions, a negligible signal was detected, confirming that the observed fluorescence signal was specifically associated with the Nile Red staining of lipid droplets.

### 2.2. Expression of Lipid Metabolism and Osteogenic Genes

**Figure 2:**
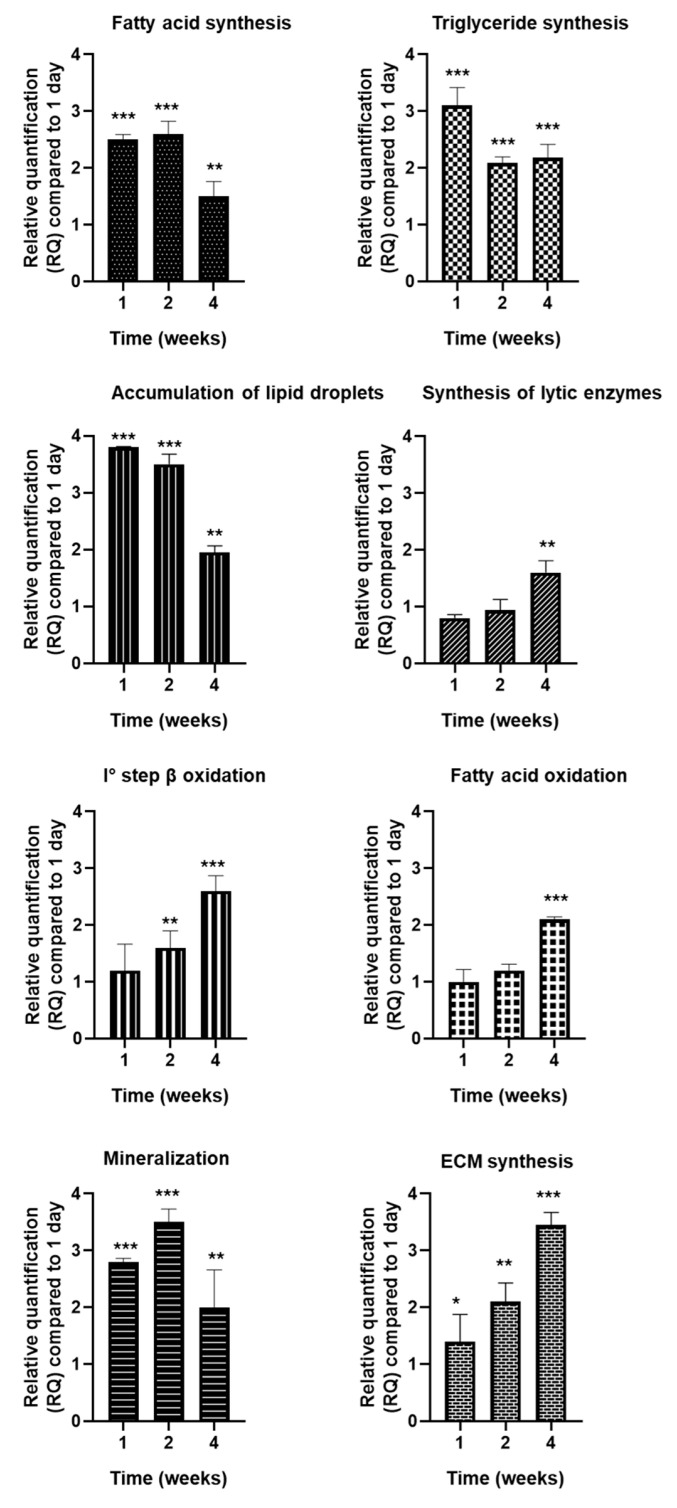
qRT-PCR analysis of lipid metabolism and osteogenic markers in 3D human osteoblast spheroids. Relative mRNA levels (*FASN*, *DGAT2*, *PLIN2*, *PNPLA2*/*ATGL*, *CPT1A*, *HADHA*, *ALPL*, and *SPARC*) are expressed as fold change ± SD normalized to day 1 and to *GAPDH*. The data represent three independent experiments performed in triplicate. Statistical significance: * *p* < 0.05, ** *p* < 0.01, *** *p* < 0.001.

Gene expression levels were normalized to day 1 of culture and are expressed as fold change ± SD relative to this baseline (Figure 2).

Genes associated with de novo fatty acid synthesis showed variation over time. *FASN* expression was 2.5 ± 0.09-fold at 1 week, slightly increased to 2.6 ± 0.23-fold at 2 weeks, and decreased to 1.5 ± 0.26-fold at 4 weeks. Similarly, genes associated with triglyceride synthesis varied over time. *DGAT2* expression was 3.1 ± 0.32-fold at 1 week, decreased to 2.09 ± 0.11-fold at 2 weeks, and remained at 2.18 ± 0.24-fold at 4 weeks. Genes associated with lipid droplet accumulation were higher at early time points. *PLIN2* was 3.8 ± 0.01-fold at 1 week, slightly reduced to 3.5 ± 0.18-fold at 2 weeks, and further decreased to 1.95 ± 0.12-fold at 4 weeks. In contrast, genes associated with lipolytic activity increased over time. *PNPLA2/ATGL* expression was 0.80 ± 0.06-fold at 1 week, increased to 0.94 ± 0.19-fold at 2 weeks, and further to 1.60 ± 0.21-fold at 4 weeks. Genes associated with mitochondrial fatty acid oxidation increased over time. *CPT1A* expression increased from 1.2 ± 0.5-fold at 1 week to 1.6 ± 0.3-fold at 2 weeks and reached 2.6 ± 0.3-fold at 4 weeks. Similarly, *HADHA* expression increased from 1.0 ± 0.2-fold at 1 week to 1.2 ± 0.1-fold at 2 weeks and further to 2.1 ± 0.1-fold at 4 weeks. Genes associated with osteogenic activity also varied over time. *ALPL* expression increased from 2.8 ± 0.1-fold at 1 week to 3.5 ± 0.2-fold at 2 weeks, followed by a reduction to 2.0 ± 0.7-fold at 4 weeks. In contrast, *SPARC* exhibited a progressive increase over time, rising from 1.40 ± 0.48-fold at 1 week to 2.10 ± 0.33-fold at 2 weeks and reaching 3.45 ± 0.22-fold at 4 weeks.

### 2.3. Raman Spectroscopy Characterization

**Figure 3:**
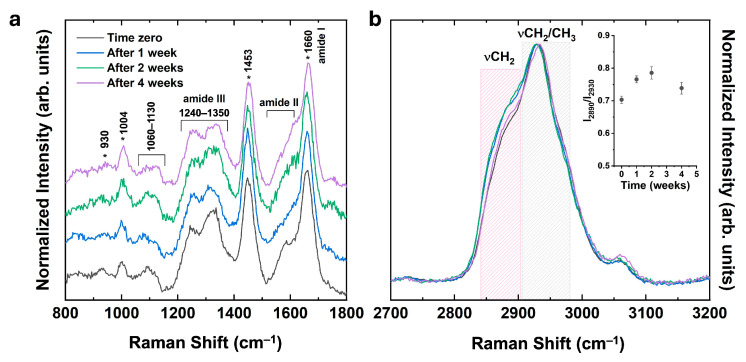
Normalized Raman spectra in staked view of the 3D human osteoblast spheroids in the protein fingerprint region (**a**) and in the high-frequency region (**b**). The inset shows the variation of the ratio between the Raman intensities at 2890 cm^−1^ and 2930 cm^−1^ (I_2890_/I_2930_) over time.

A spectroscopic characterization of 3D human osteoblast spheroids was performed using Raman spectroscopy (Figure 3) to investigate biochemical changes during spheroid maturation.

All Raman spectra were acquired in the spectral range between 800 and 3200 cm^−1^ at various time points ranging from 1 to 4 weeks, to capture the complete vibrational pattern of the samples. In Figure 3a, the Raman spectra of the 3D spheroids in the fingerprint region between 800 and 1800 cm^−1^ are shown, revealing * key peaks associated with the typical vibrational features of biological systems [22,23], as detailed in Table 1. The peak at 1004 cm^−1^ corresponds to the aromatic ring breathing mode characteristic of the phenylalanine amino acid [24]. The peak at 1250 cm^−1^ is assigned to Amide III vibrations [25], while the group of peaks between 1340 cm^−1^ and 1455 cm^−1^ is associated with the typical CH bending and deformation modes, including CH_2_ twisting, CH_2_ rocking, and CH_2_/CH_3_ scissoring [24,26]. The broad peak at 1660 cm^−1^, associated with Amide I vibrations and C=C stretching [26], indicates the presence of protein secondary structures indicative of protein backbone organization and collagen-related structures. In the high-frequency region (2700–3200 cm^−1^), lipid-associated bands were observed, particularly within the 2850–2890 cm^−1^ range, attributed to CH_2_ stretching of lipid acyl chains and the 2900–2930 cm^−1^ region associated with CH_2_/CH_3_ stretching modes [27,28]. The intensity of the lipid-related CH_2_ stretching band changed during spheroid maturation. The insert of Figure 3b highlights the temporal evolution of the intensity ratio between the 2890 cm^−1^ and the 2930 cm^−1^ bands (I_2890_/I_2930_). This ratio initially rises from early to intermediate stages, followed by a decrease at 4 weeks, suggesting time-dependent variation in lipid-associated spectral features. In parallel, progressive modulation of Amide I and Amide III bands was observed, suggesting changes in protein-related spectral features during spheroid maturation. The coordinated evolution of lipid- and protein-associated vibrational features is consistent with time-dependent changes in biochemical composition during spheroid development.

## 3. Discussion

Bone formation is a highly demanding biosynthetic process requiring sustained extracellular matrix (ECM) synthesis and mineral deposition [32,33]. While glucose metabolism has traditionally been considered the principal energetic source for osteoblasts, accumulating evidence indicates that lipid metabolism represents a relevant component of osteoblast differentiation. In this context, the present study examined the temporal coordination of lipid storage and utilization pathways in a controlled three-dimensional (3D) human osteoblast spheroid model.

Confocal imaging demonstrated that intracellular lipid distribution in 3D osteoblast spheroids underwent progressive spatial remodeling during maturation. Early stages were characterized by discrete lipid vesicles distributed within individual cells, whereas later stages exhibited compartmentalized organization, including peripheral lipid enrichment and structural reorganization within the spheroid core. The emergence of spatial heterogeneity at advanced stages further indicates that 3D architecture, with its intrinsic gradients of nutrients and oxygen, can influence lipid trafficking and metabolic adaptation in ways not observed in conventional 2D systems.

Gene expression analysis revealed a stage-dependent sequence of lipid metabolic events consistent with the morphological findings. During early spheroid maturation (1 week), the transcriptional profile was characterized by activation of pathways involved in fatty acid synthesis and triglyceride assembly, together with stabilization of intracellular lipid stores, consistent with a predominance of lipid accumulation processes during initial structural consolidation. At 2 weeks, this storage-oriented metabolic configuration remained evident, concomitant with peak osteogenic activity, suggesting increased biosynthetic and matrix-related demands. By 4 weeks, a coordinated transcriptional shift toward genes involved in lipid mobilization and mitochondrial fatty acid oxidation became apparent, suggesting enhanced utilization of stored fatty acids to sustain energy requirements during advanced spheroid maturation. This temporal transcriptional shift from lipid accumulation-associated pathways toward lipid mobilization-related pathways is consistent with previous observations showing that lipolysis derived fatty acids support mitochondrial bioenergetic function and osteoblast activity during bone formation [5,34].

Raman spectroscopy provided complementary spectroscopic information on these molecular trends. A time-dependent variation of lipid-associated CH_2_/CH_3_ stretching bands suggests changes in lipid-related molecular composition during spheroid maturation. Parallel, progressive changes in Amide I–III regions suggest modifications in protein-related spectral features during spheroid maturation. The convergence of spectroscopic signatures with gene expression data suggests a coordinated remodeling of lipid-related molecular features and extracellular matrix organization in this 3D system [35].

Consequently, the reproducible model provides a controlled platform for studying how alterations in lipid flux and oxidative metabolism influence bone formation activity. Given the recognized association between metabolic dysfunction and skeletal disorders, including osteoporosis and metabolic bone disorders, this system may represent a valuable experimental tool for studying energy-related bone diseases. In particular, the temporal coordination between lipid storage and lipid mobilization observed in this study may be used to investigate how alterations in lipid metabolism affect osteoblast differentiation and bone formation efficiency. This model may, therefore, be applied to evaluate metabolic conditions characterized by altered lipid handling and to test metabolic interventions targeting osteoblast function by modulating lipid synthesis, lipid mobilization, or mitochondrial fatty acid oxidation.

Although 3D spheroid models do not fully reproduce the complexity of in vivo bone tissue, they represent a reproducible and controlled experimental system that allows the investigation of cell–cell interactions, spatial organization, and metabolic regulation under defined conditions. In this context, the 3D spheroid model provides a complementary approach to more complex systems such as organoids, which may represent an important direction for future studies aimed at further recapitulating tissue-level architecture.

A limitation of the present study is the lack of functional perturbation approaches, such as gene silencing or knockdown strategies (e.g., siRNA). Future studies integrating these approaches, together with protein-level validation (e.g., immunoblot analysis), will further clarify the causal contribution and functional regulation of lipid metabolism pathways during osteoblast maturation in this 3D model.

## 4. Materials and Methods

### 4.1. Cell Culture Conditions

The human fetal osteoblast cell line hFOB 1.19 (ATCC, Manassas, VA, USA) was cultured in a 1:1 mixture of Ham’s F12 and Dulbecco’s Modified Eagle Medium (DMEM/F12; D8437, Sigma-Aldrich, St. Louis, MO, USA) (D8437, Sigma/Merck Life Science), supplemented with 2.5 mM L-glutamine (G7513, Sigma-Aldrich, St. Louis, MO, USA), 0.3 mg/mL G418 (4727878001, Merck Life Science, Darmstadt, Germany), and 10% fetal bovine serum (FBS; F7524, Sigma-Aldrich, St. Louis, MO, USA). Cells were maintained at 37 °C in a humidified atmosphere with 5% CO_2_ [36]. The culture medium was replaced twice per week, and cells were subcultured at ~80% confluence. Cells were routinely tested for mycoplasma contamination and confirmed to be mycoplasma-free.

### 4.2. 3D Spheroid Formation

The 3D spheroid models were generated using the hanging drop method, as previously published [21]. Spheroid formation and growth were monitored at defined time points (1, 2, and 4 weeks). Each experimental condition was performed in triplicate and repeated in three independent experiments (*n* = 3) to ensure reproducibility.

### 4.3. Confocal Microscopy and Lipid Staining

Lipid droplets were stained using Nile Red, a fluorescent dye specific for hydrophobic lipid environments. A 30 mM Nile Red stock solution was prepared in dimethyl sulfoxide (DMSO) and diluted 1:3000 in DMEM/F12 Ham’s medium immediately before use. Spheroids were gently transferred onto glass slides using sterile forceps and washed twice with Dulbecco’s Phosphate-Buffered Saline (DPBS) [37]. Samples were fixed with 4% formalin for 15 min at room temperature, followed by incubation with Nile Red for 30 min at room temperature in the dark. After staining, samples were washed twice with DPBS to remove excess dye, mounted on glass slides, and analyzed using a Leica DMIRE2 inverted microscope equipped with a TCS SP2 confocal system. Confocal microscopy was used to evaluate intracellular lipid accumulation in 3D spheroids at baseline (time zero) and after 1, 2, and 4 weeks of culture. Confocal imaging was used to assess the spatial distribution of intracellular lipid droplets within the spheroid architecture during culture. For the analysis, the fluorescence excitation was performed at 514 nm, and the emission was collected at 550–600 nm to exclude the laser reflection [38]. Images were acquired at 45× magnification, and the fluorescence analysis was performed using ImageJ software v1.53 (NIH, Bethesda, MD, USA).

### 4.4. Quantitative Real-Time PCR (qRT-PCR)

The expression of genes involved in lipid metabolism, mitochondrial fatty acid oxidation, and osteogenic differentiation was analysed during 3D spheroid development at defined time points (1, 2, and 4 weeks) [39,40,41]. Total RNA was extracted from 3D spheroids using TRIzol reagent (Invitrogen, Carlsbad, CA, USA) according to the manufacturer’s instructions and quantified using a NanoDrop ND-1000 UV spectrophotometer (Thermo Fisher Scientific, Waltham, MA, USA). Complementary DNA (cDNA) was synthesized from 1 µg total RNA using a reverse transcription kit (Promega, Madison, WI, USA; Cat. No. A3800), and quantitative real-time PCR (qRT-PCR) was performed using SsoAdvanced Universal SYBR Green Supermix (Bio-Rad Laboratories, Hercules, CA, USA) in a final reaction volume of 20 μL containing 1 μL of cDNA, 0.5 μM of forward and reverse primers, and 10 μL of SYBR Green Supermix. Gene expression levels were calculated using the 2^−ΔΔCt^ method, normalized to GAPDH as the housekeeping gene, and expressed as fold changes relative to day 1. Primer sequences used for the qRT-PCR analysis are reported in Table 2.

### 4.5. Raman Spectroscopy

For the Raman experiments, all the analysed samples were transferred onto commercial CaF_2_ slides, which were selected because they minimize substrate-related fluorescence background [29] compared with glass or silica-based slides. After deposition, the samples were washed with phosphate-buffered saline (PBS), fixed with 4% formalin for 20 min, and finally rinsed with ultrapure water before the spectroscopic analysis. Raman spectra were acquired by focusing 0.5 mW of a 473 nm laser line (COBOLT) through a 100× objective with a numerical aperture of 0.9 mounted on an Olympus microscope. The backscattered signals were collected over 10 s by a Horiba iHR550 spectrometer equipped with a 600 lines/mm diffraction grating and a CCD detector (Syncerity) from Horiba. Data analysis was performed using the LabSpec 6 (version 6.1.180 beta) software (Horiba Scientific, Kyoto, Japan).

### 4.6. Statistical Analysis

All experiments were conducted in technical triplicate and performed in three independent biological replicates. Results are expressed as mean ± standard deviation (SD). Data were analysed using GraphPad Prism 8.0 (GraphPad Software, San Diego, CA, USA). Statistical significance was determined using one-way ANOVA followed by a Bonferroni post-hoc test for multiple comparisons. Statistical significance was set at a threshold of 0.05.

## 5. Conclusions

This study describes the coordinated transcriptional modulation of genes involved in lipogenic, lipolytic, and mitochondrial fatty acid oxidation pathways associated with the energetic and biosynthetic demands of osteoblast maturation and bone matrix formation. Combining 3D morphology, intracellular lipid visualization, gene expression profiling, and Raman spectroscopy, our findings indicate dynamic molecular remodeling of lipid handling pathways involving lipid droplets.

These data could contribute to the development of advanced experimental platforms for studying metabolic alterations associated with bone diseases, including conditions characterized by altered osteoblast activity and bone remodeling, such as osteoporosis and metabolic bone disorders. Furthermore, this model may represent a useful experimental platform for investigating lipid-related metabolic pathways involved in osteoblast function and for testing metabolic modulators under controlled experimental conditions.

## Figures and Tables

**Table 1:** Raman peak assignments in 3D human osteoblast spheroids.

Frequency (cm^−1^)	Vibrational Mode	Assignment	References
930	C–C stretching	Proteins, Lipids	[24,26]
1004	C=C stretching	Proteins (Phenylalanine ring breathing mode)	[21,29]
1060–1130	C–N stretching C–C rocking	Proteins	[21,30]
1240–1350	CH_2_ twisting Amide III (α-helix, β-sheet)	Proteins, Lipids	[26,29]
1455	CH_2_ scissoring CH_2_ rocking	Proteins, Lipids	[24,29]
1550–1620	Amide II C=C stretching (aromatic ring) Amide I (antiparallel β-sheet)	Proteins	[26,31]
1660	Amide I (α-helix)	Proteins	[24,26]
2850–2890	CH_2_ stretching	Lipids	[31]
2900–2930	CH_2_ and CH_3_ stretching	Lipids	[31]

**Table 2:** Gene target used in qRT-PCR.

Protein Name	Target Gene	Forward	Reverse
Glyceraldehyde3-phosphate dehydrogenase	*GAPDH*	GTCTCCTCTGACTTCAACAGCG	ACCACCCTGTTGCTGTAGCCAA
Fatty Acid Synthase	*FASN*	TTCTACGGCTCCACGCTCTTCC	GAAGAGTCTTCGTCAGCCAGGA
Diacylglycerol O-Acyltransferase 2	*DGAT2*	GCTACAGGTCATCTCAGTGCTC	GTGAAGTAGAGCACAGCGATGAG
Perilipin 2	*PLIN2*	GATGGCAGAGAACGGTGTGAAG	CAGGCATAGGTATTGGCAACTGC
Adipose Triglyceride Lipase	*PNPLA2/ATGL*	CCCACTTCAACTCCAAGGACGA	GCAGGTTGTCTGAAATGCCACC
Carnitine Palmitoyltransferase 1A	*CPT1A*	GATCCTGGACAATACCTCGGAG	CTCCACAGCATCAAGAGACTGC
Hydroxyacyl-CoA dehydrogenase trifunctional multienzyme complex subunit alpha	*HADHA*	GCCGACATGGTGATTGAAGCTG	GGAGAGCAGATGTGTTACTGGC
Secreted protein acidic and cysteine-rich	*SPARC*	TGCCTGATGAGACAGAGGTGGT	CTTCGGTTTCCTCTGCACCATC
Alkaline Phosphatase, Biomineralization Associated	*ALPL*	GCTGTAAGGACATCGCCTACCA	CCTGGCTTTCTCGTCACTCTCA

## Data Availability

All data supporting the findings of this study are available within the document.

## Abbreviations

The following abbreviations are used in this manuscript:

3D	Three-dimensional
ALPL	Alkaline phosphatase, tissue-nonspecific isozyme
ATGL	Adipose triglyceride lipase
CPT1A	Carnitine palmitoyltransferase 1A
DGAT2	Diacylglycerol O-acyltransferase 2
DMEM	Dulbecco’s Modified Eagle Medium
DPBS	Dulbecco’s phosphate-buffered saline
ECM	Extracellular matrix
FASN	Fatty acid synthase
FBS	Fetal bovine serum
GAPDH	Glyceraldehyde-3-phosphate dehydrogenase
HADHA	Hydroxyacyl-CoA dehydrogenase trifunctional multienzyme complex subunit alpha
PBS	Phosphate-buffered saline
PLIN2	Perilipin 2
PNPLA2	Patatin-like phospholipase domain-containing protein 2
qRT-PCR	Quantitative real-time polymerase chain reaction
SD	Standard deviation
SPARC	Secreted protein acidic and cysteine rich
